# Human-influenced diets affect the gut microbiome of wild baboons

**DOI:** 10.1038/s41598-023-38895-z

**Published:** 2023-07-23

**Authors:** Madelyn Moy, Laura Diakiw, Katherine R. Amato

**Affiliations:** 1 Department of Anthropology, Northwestern University, Evanston, IL 60208 USA; 2 Department of Ecology, University of Wyoming, Laramie, WY 82071 USA

**Keywords:** Symbiosis, Microbiome

## Abstract

Industrialized diets that incorporate processed foods and are often high in simple sugars and fats and low in fiber have myriad health impacts, many of which may operate via impacts on the gut microbiota. Examining how these diets affect the gut microbiota can be challenging given that lab animal models experience altered environmental contexts, and human studies include a suite of co-varying cultural and environmental factors that are likely to shape the gut microbiota alongside diet. To complement these approaches, we compare the microbiomes of wild populations of olive baboons (*Papio anubis*) with differential access to human trash high in processed foods, simple sugars, and fats in Rwanda’s Akagera National Park. Baboons are a good model system since their microbiomes are compositionally similar to those of humans. Additionally, this population inhabits a common environment with different social groups consuming qualitatively different amounts of human trash, limiting variation in non-dietary factors. Using 16S rRNA gene amplicon sequencing we find that baboons with unlimited access to human trash have reduced microbial alpha diversity and reduced relative abundances of fiber-degrading taxa such as *Ruminococcaceae*, *Prevotellaceae*, and *Lachnospiraceae*. In contrast, baboons with limited access to human trash have a microbiome more similar to that of baboons with no access to human trash. Our results suggest that while a human-influenced diet high in processed foods, simple sugars, and fats is sufficient to alter the microbiome in wild baboons, there is a minimum threshold of dietary alteration that must occur before the microbiome is substantially altered. We recommend that data from wild primate populations such as these be used to complement ongoing research on diet-microbiome-health interactions in humans and lab animal models.

## Introduction

Industrialized diets that are high in processed foods, simple sugars, and fats and low in fiber are reported to have a range of negative health impacts in humans and other animals^1^. There are multiple mechanisms through which these effects can operate, but a recent pathway of interest is the gut microbiota. The gut microbiota is known to influence host metabolism, immune function, and behavior^2–4^, and its composition and function are strongly shaped by host environmental factors, including diet. In fact, variation in diet is one of the strongest predictors of variation in the gut microbiota for a given host species.

Studies of lab animal models have provided strong causal evidence that low-fiber and high-fat diets are associated with marked shifts in the gut microbiota. For example, when mice are provided a low-fiber diet, they exhibit an intergenerational decline in gut microbiome diversity as reduced availability of carbohydrate substrates leads to the loss of microbial taxa that degrade them^5^. Similarly, rhesus macaques consuming a ‘Western’ diet that is low in fiber and high in fat exhibit reduced microbial diversity as well as increased relative abundances of taxa such as *Ruminococcus* and *Coprococcus* and decreased relative abundances of taxa such as *Lactobacillus, Clostridium, Faecalibacterium,* and *Oscillospira*^6^. Finally, a recent meta-analysis of 27 gut microbiome studies found that high-fat diets consistently induced a high ratio of Firmicutes to Bacteroidetes as well as increased relative abundances of microbial taxa such as *Lachnospiraceae*, *Ruminococcaceae*, and *S24-J Muribaculaceae.* While these studies are critical for identifying potential diet-microbe interactions, laboratory animals’ physical and social environments are drastically altered compared to what they would experience in the wild, and these factors can alter host-microbe dynamics. For instance, mice deprived of dietary fiber may lose microbial taxa and functions permanently in a controlled laboratory experiment, while environmental microbial transmission could dampen this effect in the wild.

Experimental studies in humans tend to fall on the other extreme. Environments and lifestyles are often uncontrolled, leading to substantial inter-individual variation in a range of factors that can introduce noise into microbiome data. It is perhaps not surprising, therefore, that most human diet intervention studies have produced mixed results. For example, one study in which participants consumed a no-fiber/high-fat diet (in the form of only meat and cheese) for one week reported marked differences in microbiome composition, but not alpha diversity^7^. Another study with a no-fiber intervention reported analogous results^8^. Similarly, a study in which participants were fed either a high-fiber/low-fat or a low-fiber/high-fat diet over ten days reported only very small differences in microbiome composition, and not alpha diversity^8^. Another three-week high-fiber diet intervention also reported small shifts in gut microbiome composition, particularly an increased relative abundance of *Bifidobacterium*^9^*.*

Observational data describing microbiome differences across human populations provide more consistent findings in that ‘industrialized’ populations in high-income nations with diets rich in processed foods almost always exhibit reduced microbial alpha diversity compared to ‘non-industrialized’ populations^10–17^. Additionally, industrialized populations generally have lower prevalence and relative abundances of fiber-degrading taxa from three main families: Prevotellaceae, Spirochaetaceae, and Succinivibrionaceae^18^. In contrast, industrialized populations tend to have higher relative abundances of mucin specialists such as *Akkermansia* as well as more oxygen tolerant taxa such as Enterobacteriaceae^11,18^. These patterns are often used to argue that the low fiber content of industrialized diets leads to the loss of microbial taxa. Nevertheless, most comparisons of industrialized and non-industrialized populations do not sample people within the same geographical vicinity and therefore include substantial environmental and cultural differences beyond diet, including antibiotic use, GI parasite, and environmental exposure^19–21^. These differences represent potential confounds given that many of them also affect the gut microbiota^20,22,23^.

In this context, studies of wild animals exposed to human diets that are high in processed foods represent an important tool for diet-microbiome studies that can overcome some of the limitations of lab animal and human studies. Specifically, host physical and social environments are not artificially controlled, but inter-individual and inter-population differences in non-dietary environmental factors are relatively small^24,25^. However, despite the potential utility of data from these study systems, they are are currently underrepresented in the literature.

To begin to address this gap, we use a wild population of olive baboons with differential access to human-derived foods in garbage dumps in Rwanda’s Akagera National Park to explore the effect of a human diet on the gut microbiome. Recent research has demonstrated that the human gut microbiome is more similar to that of baboons than chimpanzees, despite our closer phylogenetic relationship to chimpanzees^26^. Therefore, baboons represent a powerful model system for addressing these questions. Additionally, human-derived foods consumed by baboons from garbage dumps are often derived from processed human foods high in sugars and fats and low in fiber^27,28^, and creating a powerful natural diet experiment. The study of so-called ‘garbage dump baboons’ dates back to the 1980s, when studies demonstrated that baboons with access to human trash tend to be more sedentary, spending less time feeding and more time resting, when compared to their wild-feeding counterparts^29^. ‘Garbage dump baboons’ also have increased body weight and subcutaneous fat thickness, and they exhibit elevated leptin levels and insulin resistance, all of which are associated with metabolic disease in humans^30–32^. However, to our knowledge, no study has compared the gut microbiomes of ‘garbage dump baboons’ to those of nearby wild-feeding counterparts.

Here we analyze the gut microbiomes of baboons with varying access to human trash (unlimited, limited, and none) to test the hypothesis that humanized diets high in processed foods are associated with changes in the taxonomic composition of the baboon gut microbiome. Specifically, we predict that baboons with access to human trash will have reduced microbial alpha diversity and reduced relative abundances of microbial taxa associated with fiber degradation and SCFA production such as *Prevotellaceae, Spirochaetaceae, Succinivibrionaceae, Fusobacteraceae, Lachnospiraceae,* and *Ruminococcaceae*. We also predict that baboons with unlimited access to human trash will exhibit more marked differences in microbiome composition than baboons with limited access. These data will provide insight into how a diet derived from processed human foods can influence the baboon gut microbiota and will contribute to our broader understanding of host-gut microbiota-diet interactions in humans and other animals occupying a range of environments with different dietary exposures.

## Methods

### Field site

Akagera National Park (ANP; 1°52’S, 30°42E) lies in eastern Rwanda, along the border with Tanzania. It is roughly 250,000 ha in size and is characterized by woodland, swamps, low mountains and savannah. At least one quarter of the park contains the swamps and lakes of the Kagera River system, and a further 5000 ha are settled. The rest of the park is composed of rolling hills with altitudes that range from 1250 to 1825 m (WCS, 2008) as well as acacia savanna with open grasslands and flooded plains in valleys and near Lake Ihema (the eastern part of ANP).

There are two lodge sites with permanent structures in the southern part of the park, one hotel and one tented camp. There is also a ranger station at the north end of the park. These three sites allow baboon access to human-derived foods in the form of trash, although the tented camp and ranger site are well protected and rarely have baboons accessing their small trash sites. The hotel has a large dumpster area where baboons typically have unlimited access to trash.

### Study groups

**Figure 1:**
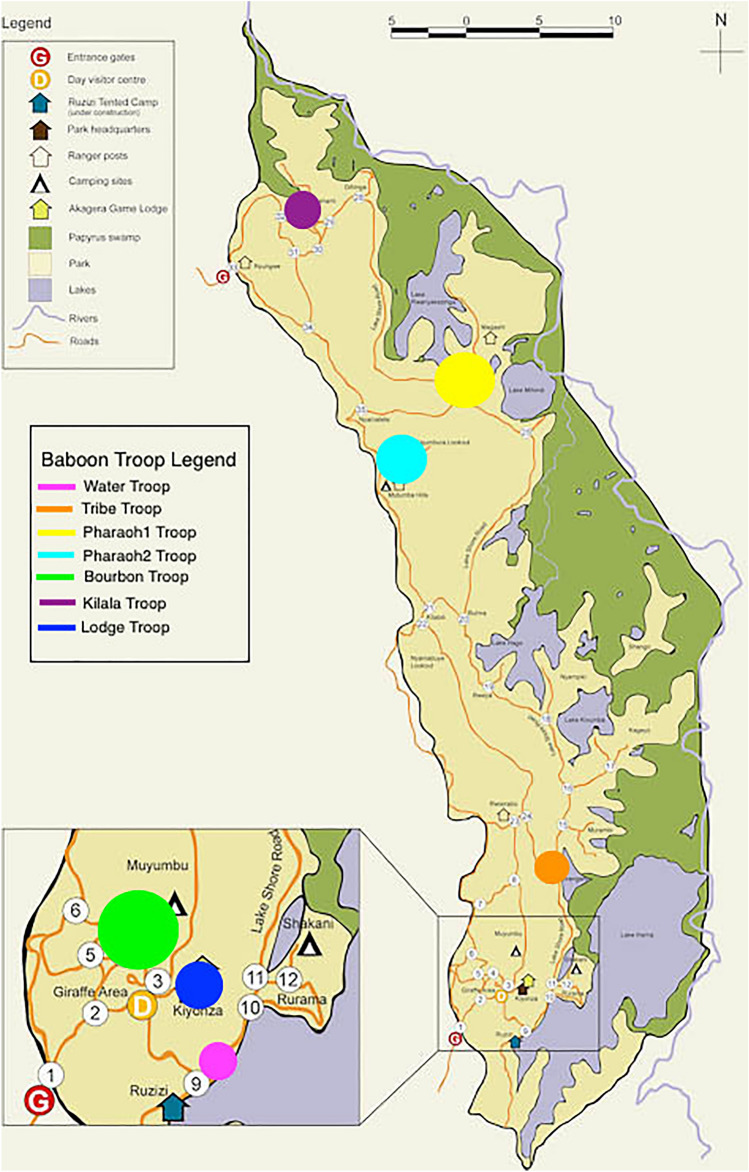
Map of Rwanda’s Akagera National Park with Approximations of Each Baboon Troop’s Range. The original map was provided by park management during the study and can currently be found here: http://www.handzaround.com/journal-1/rwandas-akagera-national-park-the-most-affordable-unique-safari-in-east-africa. Troop locations were added using Adobe software.

We included seven baboon groups in this study that ranged from habituated to semi-habituated to non-habituated (Fig. 1, Table S1). Observations suggest that group sizes ranged from 24 to 41 individuals. However, complete demographics could not be collected from unhabituated groups, so group size could be larger in some cases. One study group had territory directly adjacent to the hotel (Lodge). This group had unlimited access to trash, and the diet was qualitatively observed to consist primarily of human trash, supplemented with leaves, grass, roots, bark, flowers, tubers, fruit, and seeds. The trash was sourced mainly from the hotel kitchen, which served a menu made up of primarily European and American dishes (Table S2). The other groups were located farther away from human populations and trash access. Two of these had limited access to trash (Water, Bourbon) in the form of small refuse piles created by humans that occasionally occupied the area. These groups consumed trash approximately 25% of the time they were observed, and thus had a mixed diet of human trash and leaves, grass, roots, bark, flowers, tubers, fruit, and seeds. The Water Troop also had very limited access to fish from the fishing village. Finally, three groups were not observed to have any access to trash (Pharaoh, Tribe, Kilala). These groups were qualitatively observed to consume leaves, grass, roots, bark, flowers, tubers, seeds, and fruit (occasionally). During the study, the Pharaoh group split into two separate groups, but both groups retained the same dietary patterns as the original group.

### Sample collection

Samples were collected during June/July 2016 and January/February 2018 by Laura Diakiw. During the first period the average temperatures were 14–27 °C, and rainfall was 5 mm. During the second period, the average temperatures were 15–25 °C, and rainfall was 70 mm. We collected a total of 268 samples from all seven groups over both field seasons. Five groups were sampled in both years. Because individuals were not identified in these groups, samples were collected on a single day for each group during each season to reduce the risk of collecting multiple samples from the same individual. Samples were collected within one hour after defecation and preserved with 95% ethanol. They were kept at room temperature until transport from Kigali, Rwanda, to Tucson, Arizona (LEEP, University of Arizona), where they were frozen at – 80 °C. Samples were shipped frozen to the Amato Lab at Northwestern University for processing.

### Sample processing

DNA was extracted from each sample using the Qiagen DNEasy PowerSoil kit with modifications. Briefly, samples with solution C1 were incubated at 65 °C for 15 min before vortexing for 10 min. Additionally, 100 uL per sample of solution C6 was warmed at 65 °C for 25–30 min, and samples were incubated at room temperature after the addition of the pre-warmed C6 solution. A two-step polymerase chain reaction (PCR) was used to amplify the V4 region of the 16S rRNA gene using the 515f and 926r Earth Microbiome Project primers (www.earthmicrobiome.org) that had a Fluidigm CS1 or CS2 linker sequence added to the primer sequence as described previously^33^. Extraction and PCR negatives were used to control for contamination. PCR products were purified and normalized using a SequalPrep Normalization Plate and sequenced on the Illumina MiSeq V4 platform at the University of Illinois Chicago DNA Services Facility. Raw DNA sequences are available in the Sequence Read Archive (PRJNA880866).

Sequencing yielded 6,577,649 raw sequence reads. All controls except two extraction blanks had fewer sequence reads than the actual samples. These two blanks appeared to have been affected by contamination from neighboring samples. However, our results were not substantially impacted by including or excluding the samples extracted at the same time as these blanks. Additionally, we examined the taxa in these two blanks plus one other blank that had more than 1000 reads (but less than all other samples). Using un-rarefied, normalized data, we identified 141 ASVs out of 11, 541 that had a mean in the three extraction blanks that was greater than the mean in the actual samples plus three standard deviations. Of these, 65 were not present in any real sample and are likely to be lab contaminants of some sort. All ASV detected in the blanks (whether lab contaminants or not) had an average relative abundance of less than 0.1% in the blanks except for one ASV, Pelobacteraceae (2% relative abundance in the blanks). This ASV was not present in the actual samples and therefore was likely a lab contaminant. However, it was not driving any of our differential abundance patterns. Based on this information, we excluded the controls and included all real samples in our analyses.

Excluding controls, we had an average of 61,151 sequences per sample before quality filtering (range of 30,838 to 98,539 sequences per sample). Raw sequence data were trimmed, quality-filtered, and dereplicated, amplicon sequence variants (ASVs) were inferred, and paired reads were merged using the DADA2 plug-in^34^ for QIIME2 (v2019.7)^35^. Taxonomy was assigned in QIIME2 using a Naive Bayes classifier trained on the Greengenes 13_8 99% OTU database using the full 16S rRNA gene sequence lengths. Mitochondria and chloroplast ASVs were filtered from the dataset. After quality filtering, there was an average of 22,442 sequences per sample (range: 8206–48,051).

We generated alpha rarefaction curves using the alpha-rarefaction command, and based on the output chose to discard six samples with fewer than 11,000 reads. We used the breakaway wrapper in QIIME2 to estimate the taxonomic richness of each sample, and we removed any sample with an error greater than 15 from further alpha diversity analyses (13 additional samples). We calculated the Shannon and Faith’s Phylogenetic diversity measures using the diversity plug-in in QIIME2. To generate unweighted and weighted UniFrac distance matrices describing pairwise similarity between samples, we used the core-metrics-phylogenetic command, rarefying the data to 11,267 reads per sample. Analysis code is available on GitHub (https://github.com/Kramato-lab/Rwanda_garbage_baboon).

### Statistical analysis

To test for significant differences in overall gut microbiome composition in response to diet, we ran a permutational analysis of variance (PERMANOVA) using the adonis2 function in the package, vegan^36^, for both the unweighted UniFrac and weighted UniFrac distance matrices. Because we collected data across two years, and collection year had a significant effect on gut microbiome composition when using unweighted UniFrac distances (including only groups sampled in both years; Pseudo F_1,81_ = 2.30, r^2^ = 0.03, p < 0.001), we stratified these models by year. We used a pairwise adonis test^37^ to determine which diet types drove overall differences. We also tested for the effect of social group within each diet type using PERMANOVA. We used the betadisper function in vegan to test for homogeneity of variance between groups. We evaluated differences in microbial richness and alpha diversity across diets using ANOVA. Although year was included in the models, it had no significant effect on microbial richness and diversity. Finally, to test for differences in the relative abundance of individual GM taxa across diet types we used a series of linear regressions on rarefied relative abundance data, accounting for year. We corrected the resulting P-values for multiple tests (fdrtool). We repeated this process at the genus, family, and phylum level. We also used the ANCOMBC package^38^ to test for significant differences in the relative abundances of individual GM taxa using the ancombc2 function. R version 4.2.2 was used for all analyses. Analysis code is available on GitHub (https://github.com/Kramato-lab/Rwanda_garbage_baboon). The ASV table, metadata, and taxonomy assignments are included in the supplemental material (Tables S3–S5).

### Ethics declarations

All methods were carried out in accordance with relevant guidelines and regulations and are reported in accordance with ARRIVE guidelines. All baboon fieldwork followed The American Society of Primatology Principles for Ethical Treatment of Non-Human Primates and the Code for Best Practices in Field Primatology and was approved by University of Arizona IACUC #13-470. Samples were exported with Rwanda Development Board- Tourism and Conservation Department permit /RDB-T&C/V.U/16 and imported with CDC permits 2016-05-154 and 2018-01-155.

## Results

**Figure 2:**
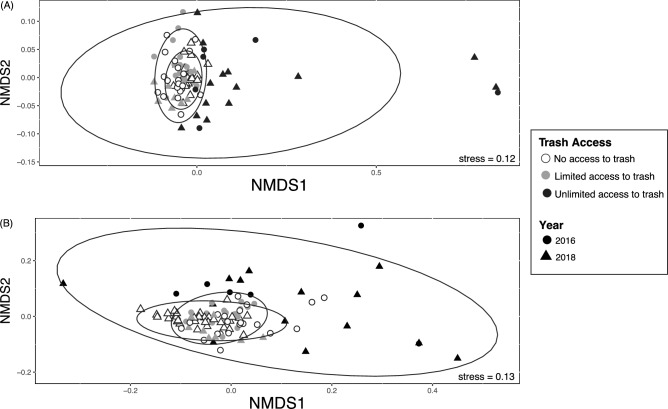
Non-metric multidimensional scaling (NMDS) plots demonstrating differences in baboon gut microbiome composition as measured using (**A**) unweighted UniFrac distances and (**B**) weighted UniFrac differences.

**Figure 3:**
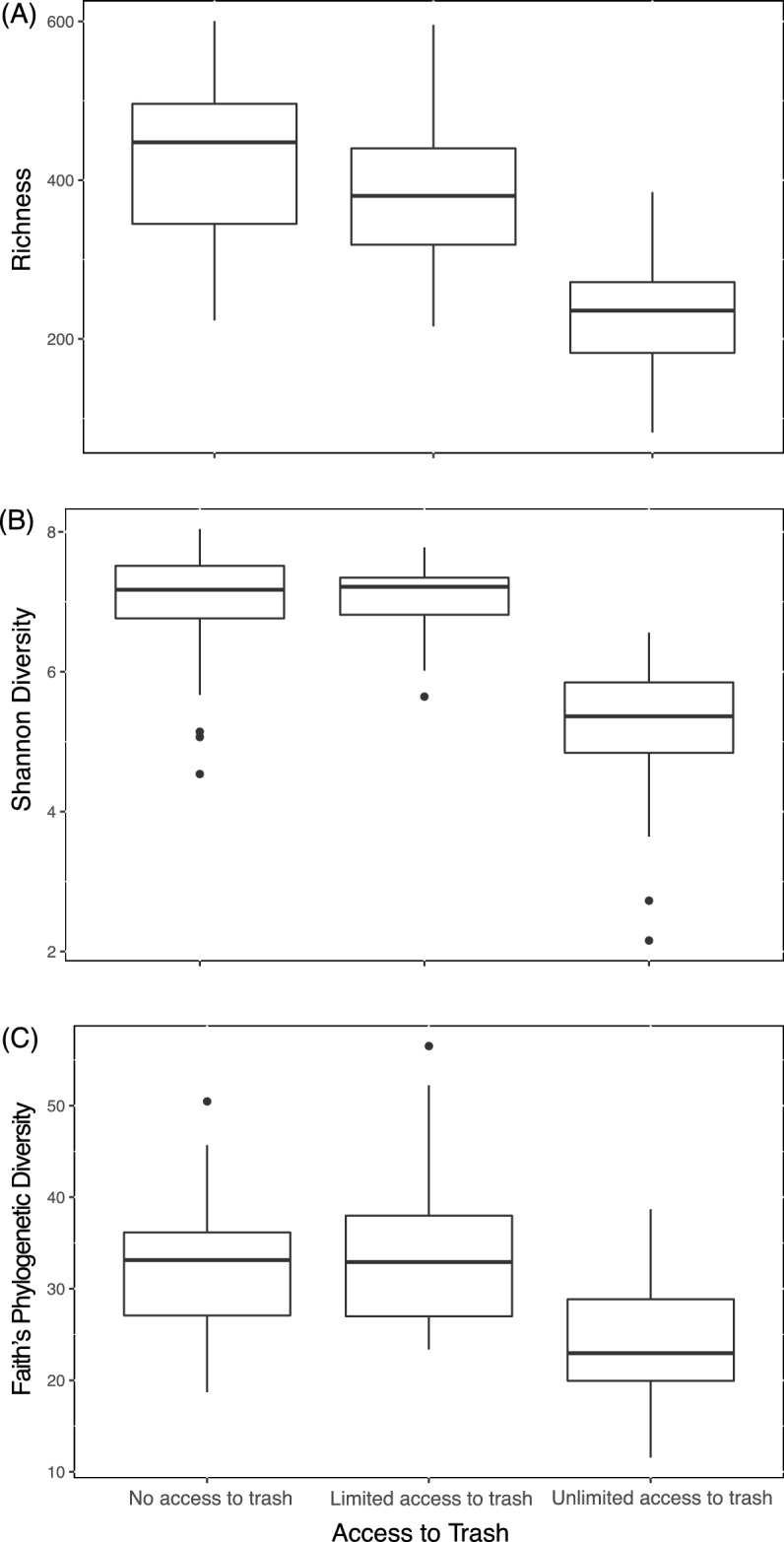
Boxplots of microbial ASV richness (**A**) and diversity (**B,C**) in response to baboon trash consumption.

**Figure 4:**
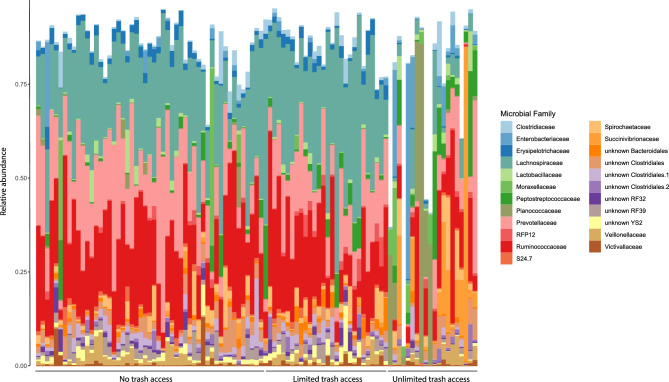
Stacked bar chart illustrating differences in the relative abundances of microbial families among baboons with no trash access, limited trash access, and unlimited trash access. Samples from all groups and sampling years are included in the plot. Only families with at least 0.5% relative abundance are included.

Overall baboon gut microbiome composition was associated with diet type (unweighted UniFrac: Pseudo F_2,98_ = 4.6, r^2^ = 0.09, p < 0.001; weighted UniFrac: Pseudo F_2,98_ = 7.5, r^2^ = 0.13, p < 0.001; Fig. 2). Specifically, baboons with unlimited access to trash had a distinct gut microbiome from baboons with limited (unweighted UniFrac: Pseudo F_1,46_ = 4.6, p = 0.001; weighted UniFrac: Pseudo F_1,46_ = 8.0, p = 0.001) and no access to trash (unweighted UniFrac: Pseudo F_1,71_ = 7.1, p = 0.001; weighted UniFrac: Pseudo F_1,71_ = 11.2, p = 0.001). Overall, gut microbiome composition was similar between baboons with limited and no access to trash (weighted UniFrac: Pseudo F_1,78_ = 1.6, p = 0.30), although analyses with unweighted UniFrac distances suggested some differences in community membership (unweighted UniFrac: Pseudo F_1,78_ = 2.0, p = 0.003) but not overall microbiome composition. Within each sampling year, there were no differences in sample dispersion in relation to trash access. When data from both years were combined, baboons with unlimited access to trash exhibited increased sample dispersion compared to baboons with limited or no access to trash (unweighted UniFrac: F_2,96_ = 19.1, p < 0.001; weighted UniFrac: F_2,96_ = 31.0, p < 0.001). Groups with unlimited access to trash had lower microbial richness (F_1,89_ = 28.4, p < 0.001) and alpha diversity (Shannon: F_1,89_ = 42.1, p < 0.001; Faith’s PD: F_1,89_ = 10.4, p < 0.001) than groups with both limited and no access to trash (Fig. 3). Additionally, our regression models indicated that baboons consuming different diet types exhibited significant differences in the relative abundances of 527 ASVs, 107 genera, 34 families, and 8 phyla (Tables S6–S9). In particular, the relative abundances of Ruminococcaceae (*Butyricicoccus, Faecalibacterium, Oscillospira*), Prevotellaceae*,* Lachnospiraceae (*Ruminococcus, Anaerostipes, Blautia, Dorea, Lachnospira, Pseudobutyrivibrio*), *Coriobacteriaceae* (*Slackia*), Erysipelotrichacea*e* (*Eubacterium, Bulleidia, Coprobacillus*), and S24-7 were all greater in baboons with no access to trash compared to baboons with unlimited access (Fig. 4). In contrast, the relative abundances of Planococcaceae (*Rumeliibacillus*), Succinibivrionaceae (*Succinivibrio*), Enterobacteriaceae*,* Peptostreptococcaceae, Sphingobacteriaceae (*Sphingobacterium*), Bifidobacteriaceae (*Bifidobacterium*), and Leuconostocaceae (*Weissella*) were all higher in baboons with unlimited access to trash compared to baboons with no access (Fig. 4). Relative abundance patterns seen in baboons with limited access to trash typically more closely approximated baboons with no access to trash compared to baboons with unlimited access. No individual taxa differed markedly in relative abundance between baboons with no access to trash and baboons with limited access, although there was a trend for reduced relative abundances of fiber degrading taxa such as *Prevotellaceae* (*Prevotella*) and *Ruminococcaceae* (*Faecalibacterium*) in baboons with limited access to trash.

ANCOM analysis indicated significant differences in the relative abundances of fewer taxa across diet groups. Specifically, there 39 ASVs, 12 genera, and 6 families differed in relative abundance globally across the three groups (Tables S10–S12). The majority of these taxa differed in relative abundance primarily between baboons with no access to trash and baboons with unlimited access to trash. Therefore, although a smaller subset of taxa were identified as contributing to the overall patterns in microbiome composition, baboons with limited access to trash were still reported to be more microbially similar to baboons with no access to trash.

Within each diet type, social group had an effect on gut microbiome composition for samples collected in 2016 (limited trash— unweighted UniFrac: p < 0.05; weighted UniFrac: Pseudo F_1,18_ = 3.5, r^2^ = 0.17, p = 0.0012; no trash— unweighted UniFrac: p < 0.05; weighted UniFrac: Pseudo F_1,20_ = 2.1, r^2^ = 0.10, p = 0.04). However, given that effects were only detected using weighted UniFrac indices, it appears that they were driven primarily by differences in the relative abundances of common microbial taxa. Accordingly, we detected no differences in microbial richness or alpha diversity across social groups within a given diet type.

## Discussion

In this study, we tested the extent to which consumption of a humanized diet high in processed foods was associated with differences in the composition of the gut microbiome of wild baboons with varying access to human trash. In accordance with our hypothesis, we found that the consumption of trash was associated with reduced baboon gut microbial alpha diversity as well as reduced relative abundances of taxa involved in fiber degradation and SCFA production. However, this pattern was only observed for baboons with unlimited access to trash. Differences in baboons with only occasional access to trash were minimal. These results suggest that a humanized, processed food diet can alter the gut microbiota even when other environmental factors are held relatively constant, but that diet shifts must be of a certain magnitude or temporal frequency to have a robust effect. Future studies should incorporate quantitative diet data as well as baboon physiological data to further test the diet-microbiota-health interactions in this wild primate system.

Our data contribute to the growing literature indicating the strong effect of diet on the gut microbiota. Despite living in similar natural environments, the baboon groups we sampled had distinct access to human-derived foods in the form of trash. Previous studies of baboon nutrition have demonstrated that human-derived foods consumed by ‘garbage dump baboons’ are higher in fat and lower in fiber than natural foods^27^. While we could not generate quantitative nutritional data in this study, the processed and cooked meats and grains that were produced by the hotel kitchen (Table S2) and ultimately ended up in the trash are likely to have a higher fat and lower fiber content than the natural baboon diet. Reduced microbial diversity and reduced relative abundances of fiber degrading taxa such as *Prevotellaceae, Ruminococcaceae*, and *Lachnospiraceae* are consistently reported in studies of humans consuming high-fat, low-fiber industrialized diets^10–13,18^, and we observed the same differences between baboons with no access to trash and baboons with unlimited access to trash in our study. These results suggest that some of the same microbial extinctions hypothesized to occur in industrialized human populations consuming a diet high in processed foods, simple sugars, and fats may also occur in other primates. While we cannot isolate the effects of different dietary components on the baboon gut microbiome in this study, the observed patterns suggest that differences in fiber intake are major contributors to microbiome composition in this context. Because microbiota-accessible carbohydrates that make up dietary fiber are the primary source of energy for the distal gut microbiota, reduced fiber diets generally do not support a diverse gut microbiota, with fiber-degrading specialists often being the first taxa to disappear from the gut^5^. Our data from baboons with unlimited access to trash demonstrate this pattern clearly, and even the baboons with limited access to trash trended toward lower relative abundances of fiber degrading microbial taxa.

While many of our findings are consistent with the human and lab animal model literature linking processed food diets to microbiota shifts, some of them were not. For example, two of the main microbial families that are commonly depleted in industrialized human populations, Succinivibionaceae and Spirochaetaceae, were not depleted in baboons with access to trash and instead exhibited increased and unchanged relative abundances, respectively. We also did not detect an enrichment in microbial taxa associated with a high fat diet in baboons with either limited or unlimited access to trash^39^. Some of these inconsistencies may be a result of fine-scale differences in diet and environment across studies. However, it is also important to note that the taxonomic resolution of data in many microbiome studies is limited, and microbial families and genera detected across studies and across host species may have different functions. For instance, a recent paper comparing butyrate production genes across the primate phylogeny, including humans, showed that different microbial taxa contain butyrate production genes in different host species^40^. Therefore, it is possible that similar shifts in gut microbiota function are shared across host species and study contexts even when microbial taxonomic patterns diverge.

Our data also suggest there may be a dietary threshold that must be exceeded for diet to substantially affect the gut microbiota. Despite occasionally consuming human trash, the baboons with limited access to trash had a gut microbiome that primarily resembled that of a wild baboon consuming a natural diet. This phenomenon has not been widely explored in the literature, but it is reasonable to believe that a similar ‘dosing threshold’ could exist for processed foods in humans. Alternatively, the temporal frequency with which an processed foods are consumed could dictate the magnitude of gut microbiota change. In this study, the baboons with limited access to trash not only consumed smaller quantities of trash as a result of the size of the refuse piles available to them, but also the frequency with which these piles were available. Future studies in this and other similar primate populations could pinpoint the minimum quantity and frequency of processed food items consumed necessary to shift the gut microbiota more precisely by quantitatively measuring individual consumption of human-derived foods in conjunction with microbiome analyses. For instance, a study of rhesus macaques (*Macaca mulatta*) recently demonstrated that 80% diet similarity was sufficient to result in microbiome convergence between a semi-wild and captive population^41^.

Although the baboons in this study all inhabited the same national park, we acknowledge that other environmental factors beyond diet may also have differed slightly across groups and contributed to some of the variation in microbiome composition. For example, the baboons with unlimited access to trash sometimes ingest human hygiene products such as toothpaste or chlorinated pool water, which could increase exposure to anti-microbial substances. Additionally, there is no direct contact between baboons and humans at this site, but because the baboons with unlimited access to trash spend most of their time near the hotel, they likely have altered contact with environmental microbial pools compared to the other groups. We did not see obvious evidence of these dynamics in our data. For example, studies of captive primates indicate increased relative abundances of microbial taxa such Prevotellaceae due to human contact, but the baboons with unlimited access to trash had lower relative abundances of Prevotellaceae^42^. Factors such as different environmental and soil microbes, less contact with baboons outside of their troop, and indirect contact with human microbes all could leave to distinct patterns of microbial dispersal^43–45^. Nevertheless, these environmental differences are subtle given that all of the baboons inhabit a natural landscape, and recent research suggests that even wild-captive environmental disparities are insufficient for creating substantial differences in the primate gut microbiome if diet is held relatively constant^41^. Therefore, we believe it is most likely that the observed patterns in our study are a result of diet.

While specific gut microbial patterns have been associated with diseases such as obesity and depression^46,47^, we cannot directly link our microbiome results to variation in baboon health. The baboons with unlimited access to trash are noticeably larger than their limited and no access counterparts. However, without physiological markers, we are unable to conclusively determine whether this increased mass is a result of increased body weight or subcutaneous fat, and whether it is accompanied by other metabolic risk factors. Moving forward, biomarkers of baboon immune, metabolic, and endocrine function should be generated in conjunction with microbiome data to explore the relationship between the baboon microbiome and health. These biomarkers can be generated from non-invasive fecal and urine samples, and in some instances, darting to collect blood samples is also possible^32,48–50^. Whether the gut microbiome serves as a biomarker for health risks or is part of the causal pathway leading to those risks, these data will advance our understanding of baboon health in anthropogenic habitats. It will also strengthen our ability to use these baboon populations as a system for exploring diet-microbiota-health interactions in humans. For instance, while our data suggest a potential threshold past which diet alterations lead to gut microbiota shifts, it is also possible that there is a threshold past which gut microbiota shifts affect host health. Generating physiological data from baboon populations with varying access to human-derived foods and distinct microbiome compositions will allow us to test this hypothesis.

## Conclusions

In conclusion, a human-derived diet high in processed foods is associated with reduced microbial alpha diversity and altered microbial composition in wild baboons. Many of the taxa that are depleted in response to an industrialized diet high in processed foods, simple sugars, and fats in humans are also depleted in baboons consuming unlimited amounts of trash. These microbial traits could contribute to health risks in baboons with access to these types of diets. However, additional data are necessary to test this relationship more robustly. Furthermore, our results suggest that while diet is sufficient to alter the gut microbiota in wild baboons, there is a minimum threshold of dietary alteration that must occur before the gut microbiota is substantially altered. ‘Garbage dump’ baboons represent a useful system for testing these dynamics in a natural environment. We recommend that data from wild primate populations such as these be used to complement ongoing research on diet-microbiota-health interactions in humans and lab animal models.

## Supplementary Information

Supplementary Legends.

Supplementary Tables.

## Data Availability

Raw DNA sequences are available in the Sequence Read Archive (PRJNA880866, https://www.ncbi.nlm.nih.gov/bioproject/PRJNA880866). Analysis code is available on GitHub (https://github.com/Kramato-lab/Rwanda_garbage_baboon).
