# Supplementary material for: Human-influenced diets affect the gut microbiome of wild baboons
Source: Sci Rep. 2023 Jul 23;13:11886. doi: 10.1038/s41598-023-38895-z (PMC10363530; doi:10.1038/s41598-023-38895-z)
Supplement: Supplementary file 1 — Supplementary Legends. [file 41598_2023_38895_MOESM1_ESM.docx]

**Supplemental Material**

*Supplemental Tables (separate file)*

Table S1. Demographics and location of baboon groups sampled at Akagera National Park.

Table S2. Table S2. Examples of menu items from the lodge where baboons had unlimited access to trash.

Table S3. ASV table for baboon gut microbiome analysis, rarefied to 11, 267 reads per sample.

Table S4. Baboon gut microbiome metadata table.

Table S5. Taxonomic assignments of ASVs from baboon gut microbiome study.

Table S6. Average relative abundances (and standard deviations) of microbial amplicon sequence variants (ASVs) that differed significantly across baboon diet groups.

Table S7. Average relative abundances (and standard deviations) of microbial genera that differed significantly across baboon diet groups.

Table S8. Average relative abundances (and standard deviations) of microbial families that differed significantly across baboon diet groups.

Table S9. Average relative abundances (and standard deviations) of microbial phyla that differed significantly across baboon diet groups.

Table S10. ASVs that were differentially abundant across baboon diet groups according to ANCOM-BC tests.

Table S11. Genera that were differentially abundant across baboon diet groups according to ANCOM-BC tests.

Table S12. Families that were differentially abundant across baboon diet groups according to ANCOM-BC tests.
